# Identifying Changes of Brain Regional Homogeneity and Cingulo-Opercular Network Connectivity in First-Episode, Drug-Naïve Depressive Patients With Suicidal Ideation

**DOI:** 10.3389/fnins.2022.856366

**Published:** 2022-03-02

**Authors:** Mengxin He, Liangliang Ping, Zhaosong Chu, Chunqiang Zeng, Zonglin Shen, Xiufeng Xu

**Affiliations:** ^1^Department of Psychiatry, First Affiliated Hospital of Kunming Medical University, Kunming, China; ^2^Mental Health Institute of Yunnan, First Affiliated Hospital of Kunming Medical University, Kunming, China; ^3^Department of Psychiatry, Xiamen Xianyue Hospital, Xiamen, China; ^4^Yunnan Clinical Research Center for Mental Disorders, First Affiliated Hospital of Kunming Medical University, Kunming, China

**Keywords:** major depressive disorder, suicidal ideation, regional homogeneity, functional MRI, structure connectivity

## Abstract

**Objective:**

Adult patients with major depressive disorder (MDD) may not actively reveal their suicidal ideation (SI). Therefore, this study is committed to finding the alterations in the cingulo-opercular network (CON) that are closely related to SI with multi-imaging methods, thus providing neuroimaging basis for SI.

**Method:**

A total of 198 participants (129 MDD patients and 69 healthy controls) were recruited and evaluated with the Montgomery–Asberg Depression Rating Scale (MADRS). The healthy individuals formed the HC group, while the MDD patients were subdivided into no SI MDD (NSI, *n* = 32), mild SI MDD (MSI, *n* = 64), and severe SI MDD (SSI, *n* = 33) according to their MADRS item 10. We obtained MRI data of all participants and applied regional homogeneity (ReHo) analysis to verify a previous finding that links CON abnormality to SI. In addition, we employed the structural covariance network (SCN) analysis to investigate the correlation between abnormal structural connectivity of CON and SI severity.

**Results:**

Compared to those of the HC group, MDD ReHo values and gray matter volume (GMV) were consistently found abnormal in CON. ReHo values and GMV of the right orbital inferior frontal gyrus (ORBinf.R) in the MDD group decreased with the increase of SI. Compared to the HC group, the MDD patients showed enhanced structural connectivity of three pairs of brain regions in CON [ACC.L–left superior frontal gyrus (SFG.L), SFG.L–left middle temporal gyrus (MTG.L), and the SFG.L–left post-central gyrus (PoCG.L)]. Compared with that of the NSI and MSI groups, the structural connectivity of three pairs of brain regions in CON is enhanced in the SSI groups [ORBinf.L–right ventral posterior cingulate gyrus (VPCC.R), VPCC.R–SFG.R, and SFG.R–PoCG.R].

**Conclusion:**

Our findings showed the distinctive ReHo, GMV, and SCN pattern of CON in MDD patients with SI; and with the severity of suicide, abnormal brain regions increased. Our finding suggested that MDD patients with different severity of SI have different neuroimaging changes.

## Introduction

Major depressive disorder (MDD) is a common disease affecting more than 264 million people worldwide (World Health Organization, 2017). Depression is different from mood swings and short-term emotional responses to challenges in daily life, and long-term moderate or severe depression may become a serious illness. At its worst, depression can lead to suicide, and nearly 800,000 people die by suicide each year (World Health Organization, 2017). To a large extent, the results of the suicide risk assessment can be attributed to the subjective willingness of patients (Walker et al., 2015). Suicide incurs unacceptably high costs for society, families, and individuals (Walker et al., 2015). MDD patients with (vs. without) suicidal ideation (SI) (Cooper et al., 1994) have a higher rate of suicide attempts (Pfaff and Almeida, 2004; Du et al., 2017). SI can be considered the first step on the road to a suicide attempt (Ding et al., 2016). SI can lead to suicide attempts and may be caused by biological factors (van Heeringen et al., 2014). The degree of heritability of symptoms of depression, such as physical symptoms, guilt, and SI, has been shown to vary (h2 range, 0–35%) (Fried and Nesse, 2015). SI has a higher heritability coefficient than do other symptoms (Kappelmann et al., 2021). In addition, SI is driven by neurobiological processes; it is not merely a symptom of depression (Fried and Nesse, 2015; Du et al., 2017). Identifying SI helps to reduce suicide attempts (Ding et al., 2016).

Although the risk assessment of MDD patients can be carried out by suicide scales, an investigative study found that adults may not seek help by disclosing SI (McGillivray et al., 2022). MDD patients with mild or severe SI can develop anxiety symptoms, but people tend to pay more attention to patients with severe SI while ignoring those with mild SI. This results in poor efficacy for these patients and even severe, chronic, or even treatment-resistant depression (Lieberman et al., 2020). Therefore, studying the neural mechanisms of SI at different severity may prevent this condition. MRI has been widely used as a non-invasive method of studying brain structure and function in depressive suicide attempters (Fried and Nesse, 2015; Du et al., 2017). Neuroimaging may also be able to provide reliable indicators of SI. Elucidating the neuroimaging characteristics of SI in MDD could help clinicians to intervene early and thereby reduce the risk of suicide. While the prefrontal cortex (PFC) has been proved to be associated with SI in previous studies (Myung et al., 2016), it is generally believed that SI is related to abnormal neural network connectivity and not limited to a single brain region (Chase et al., 2017; Bani-Fatemi et al., 2018; Schmaal et al., 2020).

Recent studies have reported that disconnection of the cingulo-opercular network (CON) is the key to many mental illnesses (Jollant et al., 2010). Components of the CON include the anterior cingulate cortex (ACC), PFC, parietal cortex, and basal ganglia. These are major brain regions associated with MDD (Dosenbach et al., 2007; Kaiser et al., 2015). CON helps to flexibly control target-oriented performance and has been shown to participate in maintaining the stability of executive function cross-testing in the main cognitive control and SI processing (Dosenbach et al., 2007; Yang et al., 2020). In addition, research has suggested that key areas of the CON are related to suicide in MDD (Dosenbach et al., 2007; Bani-Fatemi et al., 2018; Yang et al., 2020). For example, it was recently reported that abnormality of brain regions in the CON is related to the severity of MDD symptoms (Sylvester et al., 2012; Rappaport et al., 2020). Since MDD patients with SI tend to have higher depression scores than those without SI, and CON is associated with disease severity, it is suggested that CON may reflect SI in MDD.

Regional homogeneity (ReHo) can reflect the temporal homogeneity of blood oxygen level-dependent signals in a certain region, revealing the temporal homogeneity of activity in various brain regions in the resting-state functional network (Yan et al., 2021). The ReHo analysis can be used to explore the neural activity of abnormal brain regions in the functional network. Structural covariance network (SCN) is an established measure of the cortex–cortex connectivity, which shows a good correspondence with transcribed brain networks and anatomical connectivity inferred from white matter fiber tract imaging (Gong et al., 2012). As a unique measure of connectivity, SCN can be used to investigate communication factors between anatomically connected non-adjacent brain regions where there is information exchange between synapses of non-adjacent neurons, forming macro-level structural covariance (Alexander-Bloch et al., 2013). In addition, SCN can provide a model for understanding progressive cortical abnormalities in mental disorders (Crossley et al., 2014). The relationship between functional ReHo and SCN changes should be explored because this information will help to increase our understanding of the mechanism of SI at the level of brain function and structural connectivity.

We propose the hypothesis that SI at different severity in MDD patients is associated with different changes of CON. ReHo value and SCN analysis method based on gray matter volume (GMV) can reflect the characteristics of CON in functional and structural connectivity and may better reveal whether the changes of CON varied with different SI severity.

## Materials and Methods

### Participants

First-episode, drug-naïve adults diagnosed with MDD were recruited from the psychiatric outpatient and inpatient departments of psychiatry in the First Affiliated Hospital of Kunming Medical University between 2015 and 2017. To mitigate the influence of vascular factors on brain structure, the participants in this study were adults under the age of 45 (Kendler et al., 2009). The psychiatric diagnosis was based on the DSM-IV, and at least two psychiatrists were in agreement (vanPraag, 1990). Hamilton Anxiety Scale (HAMA) was used to assess the severity of participants’ anxiety symptoms (Hamilton, 1960). All of the patients had been diagnosed with MDD for the first time and had never received anti-sedatives or systemic psychotherapy. An HC group was matched for age, gender, education level, and dominant hand. The exclusion criteria of MDD patients were as follows: previous brain injury with loss of consciousness, history of cortisol drug use, history of substance abuse, previous neurological disease, pregnancy, diagnosis of another mental illness or neurological disease, received electroconvulsive therapy for MDD, and dominant left hand. The exclusion criteria of the HC group were as follows: previous brain injury with loss of consciousness, pregnancy, history of psychiatric or neurological disease, and dominant left hand. The research protocol was approved by the First Affiliated Hospital of Kunming Medical University’s ethics committee. All participants were provided with details about the study, and their consent was obtained.

### Subgroups

To better study whether the brain abnormality model is related to the severity of SI, the 10th item of the Montgomery–Asberg Depression Rating Scale (MADRS) measures SI on a scale of 0–6, representing the feeling that life is not worth living, the feeling that a natural death would be welcome, suicidal thoughts, and preparations for suicide (Montgomery and Åsberg, 1979). In the past, only patients with scores ≥4 were studied (Murrough et al., 2015). A MADRS 10th item score ≥4 indicating severe SI is consistent with other suicide-related scale assessments (Montgomery and Åsberg, 1979; Murrough et al., 2015). To verify the hypothesis of this current study, we extended on previous research and MDD patients with scores from 1 to 3 and ≥4. All recruited patients were divided into three groups: no SI (NSI, 0 points), mild SI (MSI, 1–3 points), and severe SI (SSI, ≥4 points).

### Image Acquisition

Magnetic resonance (MR) images were captured by an experienced radiologist using an Achieva 3.0 Tesla MRI system (Philips, Eindhoven, Netherlands) with a 16-channel phased-array head coil. T1- and T2-weighted scans were obtained for all of the participants to rule out the presence of brain abnormalities. High-resolution three-dimensional MRI scans were acquired using a fast-spoiled gradient recalled acquisition (FSPGR) sequence with the following parameters: repetition time (TR) = 7.38 ms, echo time (TE) = 3.4 ms, matrix size = 256 mm × 256 mm, field of view (FOV) = 250 × 250 mm, flip angle = 8°, slice thickness = 0.6 mm, slices = 230 with no gap, and acquisition time = 6 min 53 s.

The functional image data at rest were obtained by using the echo-planar imaging sequence with the following parameters: TR = 2,200 ms, TE = 35 ms, flip angle = 90°, FOV = 230 mm × 230 mm, matrix size = 128 × 128 mm, slice thickness = 3.0 mm without interlayer spacing, slices = 50, scan duration time = 17 min 40 s.

### Functional MRI Preprocessing and Regional Homogeneity Calculation

Data preprocessing was performed in Matlab 2018b using the resting-state functional MRI (fMRI) analysis package (DPABI^1^); the first 10 images of the initial MRI signal were discarded to reduce the effect of pre-subject instability, head motion correction, and smoothing constraints. Participants were not displaced by more than 1.5 mm in the x-, y-, or z-axes, temporally bandpass filtered (0.01–0.08 Hz) and linearly detrended, imaging data space was normalized to Montreal Neurological Institute (MNI) space and resampled to 3 × 3 × 3 mm^3^, and 24 head motion parameters were obtained.

Regional homogeneity analysis was conducted by the software DPABI for the MDD and HC groups. The time series of a given voxel with the time series of its nearest neighbors is generated by calculating the Kendall coefficient (KCC) for each ReHo to be mapped (neighboring voxels were set as 26) (Yan et al., 2021). Normalization to ReHo was performed by dividing the KCC between voxels by the average KCC of the whole brain to reduce the effect of individual differences (Tononi et al., 1998).

### Gray Matter Volume Preprocessing

FreeSurfer 7.0 software^2^ was used to process images to estimate GMV. The pre-processing included motion correction, averaging of multiple volume T1-weighted images, stripping non-brain tissues by using hybrid watershed/cortical surface deformation, automated Talairach deformation, segmentation of gray matter tissue, subdivision of white matter boundary and smoothing, intensity normalization, gray matter boundary network identification, automatic topology correction, surface deformation, and optimal positioning of intensity gradient gray/white matter boundary. The location of the gray matter/cerebrospinal fluid boundary was used to transform the maximum intensity definition to other tissue types. The entire cortex of each participant was visually inspected, and the segmentation was manually edited for inaccuracies (Fischl, 2004). The cortex was then divided based on the Destrieux atlas (Destrieux et al., 2010). This produced a vector of estimated cortical volumes for each bilateral frontal lobe, the anterior cingulate gyrus, the posterior cingulate cortex (PCC), the thalamus, the parietal lobe, the temporal lobe, and the basal ganglia in the CON (58 brain regions) for each participant (see Figure 2A and details provided in the Supplementary Material).

**FIGURE 1:**
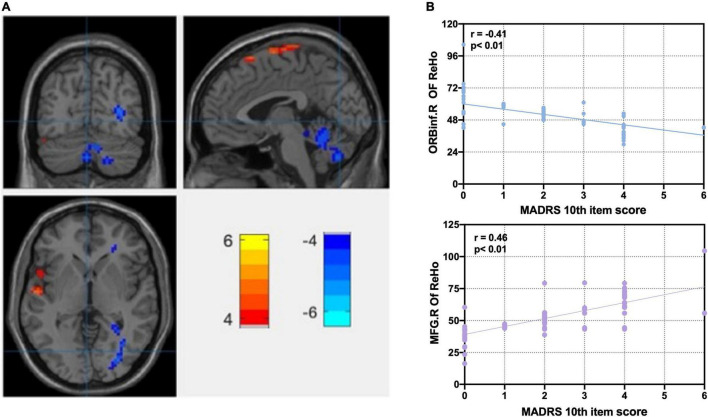
Correlation analysis of significant ReHo differences between MDD and HC groups with MADRS 10th item score. **(A)** Statistical map depicts higher and lower ReHo of MDD compared with HC groups. Abnormal ReHo of anterior cingulate cortex, prefrontal lobe, parietal cortex, and cerebellum. Disrupted regional homogeneity of the core brain region of CON at rest. **(B)** Correlation analyses between ReHo and MADRS 10th item score. Blue denotes lower ReHo, and red denotes higher ReHo. L, left side; R, right side; ReHo, regional homogeneity; ORBinf, right orbital inferior frontal gyrus; MFG, middle frontal gyrus; MADRS, Montgomery–Asberg Depression Rating Scale; FDR, false discovery rate; MDD, major depressive disorder. FDR *p* < 0.05. MADRS 10th item score: suicidal ideation, 0–6 score.

**FIGURE 2:**
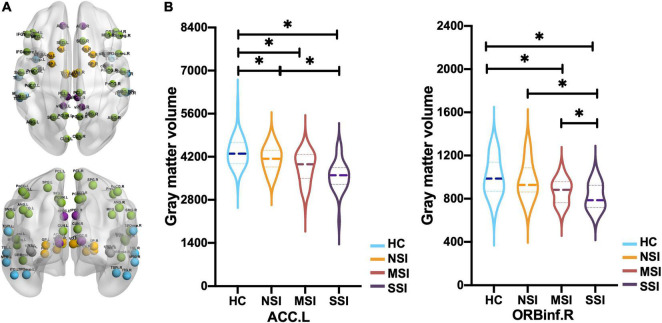
Comparison of gray matter volume (GMV) of 58 brain regions in CON. **(A)** Frontal and parietal lobe (green), cingulate gyrus (purple), temporal lobe (blue), insula (gray), basal ganglia (orange), and thalamus (gold) in CON (58 regions). **(B)** The left anterior cingulate gyrus (ACC.L) of three major depressive disorder (MDD) groups has a smaller GMV than the HC, and NSI > SSI. The right orbital inferior frontal gyrus (ORBinf.R), MSI, and SSI have smaller GMV than HC, and NSI > MSI > SSI [*p < 0.05, false discovery rate (FDR)]. Healthy group (HC), no suicidal ideation (NSI), mild suicidal ideation (MSI), and severe suicidal ideation (SSI).

### Statistical Analysis

The demographics and clinical characteristics of the participants were analyzed using SPSS 18.0. ANOVA was used to test demographic differences among three MDD groups and the HC group.

### Regional Homogeneity Analysis

A voxel-based ANOVA comparison of the whole brain ReHo maps among the MDD and HC groups was performed in REST package viewer1.8.^3^ The statistical threshold was set at *p* < 0.05 after false discovery rate (FDR) correction with an extent cluster of 100 contiguous voxels or greater using age, sex, and education level as covariates. The ReHo values of each MDD patient were extracted from the brain regions with abnormal ReHo, which are based on Matlab using the DPABI (see text footnote 1). The correlations between abnormal ReHo and MADRS total scores and 10th item scores were determined using Pearson’s correlation analyses, controlling for age, gender, and education level (*p* < 0.05).

### Gray Matter Volume Analysis

Analysis of covariance (ANCOVA) was used to test the GMV values of CON (58 brain regions) among three MDD groups and the HC group in which age, gender, and education level were used as covariates. Then *post hoc t*-tests were conducted to identify differences in the GMV values between each pair of groups by using the same covariates mentioned above. Then *post hoc t*-tests were used to compare among MDD subgroups, by using age, sex, education level, and illness duration as covariates. To correct for multiple comparisons, the FDR was controlled at 5% using the Benjamini–Hochberg procedure (Benjamini and Hochberg, 1995).

### Structural Covariance Network Analysis

Gray matter volume was used as the morphological measurement in this study, and Pearson’s correlation was used to calculate structural covariance. A Pearson’s correlation coefficient was calculated for the estimated GMV values of each pair of cortical regions. First, age, gender, and illness duration were regressed on the GMV estimates (Alexander-Bloch et al., 2013; Wannan et al., 2019). An r-to-z transformation was performed on all correlation coefficients to improve normality. Separate connection matrixes were produced for each of the three patient groups and the HC group to quantify the strength of the connection between the pair of regions. A two-sample *t-*test was used to independently test the structural covariance between the MDD groups and the control group for each area pair. A non-parametric permutation test (10,000 permutations) was used to determine the statistical significance of between-group differences in the network. Non-parametric methods were used to identify the null distribution of the data, enabling the use of non-standard test statistics (Wannan et al., 2019). FDR was controlled to correct for multiple comparisons (Benjamini and Hochberg, 1995).

## Results

### Participants’ Characteristics

Table 1 shows the demographics and clinical characteristics of the participants. There were no significant differences in gender, age, and education level between 129 patients and 69 HCs. The 198 participants were classified as follows: 32 NSI, 64 MSI, 33 SSI, and 69 HC. The four groups showed no differences in age, gender, education level, or illness duration. The NSI and MSI groups scored lower than the SSI group for total MADRS score (NSI group *t* = 1.62, *p* < 0.001; MSI group *t* = 1.37, *p* < 0.001) and MADRS 10th item score (NSI group *t* = 0.14, *p* < 0.001; ESC group *t* = 0.12, *p* < 0.001). There was no significant difference in the MADRS total scores between NSI and MSI, but both were lower than the score in the SSI group. The MSI and SSI groups scored higher than the NSI group for HAMA total score (Table 1).

**TABLE 1:** The demographics and clinical characteristics of the participants.

Variables (mean ± SD)	MDD		HC		*p*-Value
Gender (male/female)	39/90		20/49		0.13^a^
Age (years)	32.4 ± 7.6		32.9 ± 7.5		0.63^a^
Education level (years)	12.0 ± 4.3		13.0 ± 4.0		0.19^a^

**Variables (mean ± SD)**	**NSI**	**MSI**	**SSI**	**HC**	***p*-Value**

Gender (male/female)	11/21	18/46	10/23	20/49	0.73^b^
Age (years)	33.3 ± 7.7	31.9 ± 7.5	31.9 ± 7.8	32.9 ± 7.5	0.70^b^
Education level (years)	12.1 ± 3.9	12.0 ± 4.4	12.3 ± 4.1	13.0 ± 4.0	0.94^b^
MADRS total score	26.279 ± 6.0*	28.64 ± 6.3*	37.18 ± 6.7*	–	0.00^c^
MADRS 10th item (suicidal ideation)	0*	2 ± 0.61*	4.19 ± 0.59*		0.00^c^
HAMA score	18.03 ± 4.69*	24.41 ± 5.90*	25.41 ± 6.26*		0.01^c^
Illness duration (months)	14.00 ± 19.24	13.12 ± 17.81	12.39 ± 16.34	–	0.74^b^

*MDD, major depressive disorder; MADRS, Montgomery–Asberg Depression Rating Scale; HAMA, Hamilton Anxiety Scale; LSD, least significant difference.*

*^a^The p-values were obtained by two-sample t-test.*

*^b^The p-values were obtained by ANOVA.*

*^c^The p-values were obtained by chi-square test; LSD correction was used for post hoc comparison.*

*Compared to MDD groups. p < 0.05 MADRS score: SSI > NSI, MSI; HAMA score: MSI, SSI > NSI.

### Regional Homogeneity and Correlation Results

Figure 1A and Table 2 show the ANOVA of the ReHo value between the MDD and HC groups with age, gender, and education level as covariates. Compared with the HC group, there were lower ReHo values in the left PCC, the left triangular of the inferior frontal gyrus (IFGtriang), the left post-central gyrus (PoCG), the left inferior parietal gyrus (IPL), the left superior temporal gyrus (STG), the left temporal pole gyrus (TPO), and the bilateral middle temporal gyrus (MTG) in the MDD group, as well as higher ReHo values in the left ACC, the left cerebellum (CE), the right median cingulate cortex (MCC), the right middle frontal gyrus (MFG), the right orbital inferior frontal gyrus (ORBinf), and the right precentral gyrus (PreCG) in the MDD group. Disrupted ReHo is the core brain region of CON at rest (Dosenbach et al., 2007; Kaiser et al., 2015). We used the REX toolbox to extract the mean value of ReHo in different brain regions of the MDD group; after controlling for age, gender, and education level, the right orbital inferior frontal gyrus (ORBinf.R) (*r* = −0.41, *p* < 0.01) and the right middle frontal gyrus (MFG.R) were significantly correlated with SI severity (MADRS 10th item, 0–6 scores) in the MDD group (*r* = 0.46, *p* < 0.01) (Figure 1B).

**TABLE 2:** Regional homogeneity analysis between MDD and HC groups.

Cluster location HC > MDD	Hemisphere	Peak (MNI)			Number of voxels	*Z*-value
		x	y	z		
Posterior cingulate cortex, PCC	L	–6	–54	6	32	4.2727
Triangular of Inferior frontal gyrus, IFGtriang	L	–48	27	24	36	4.3395
Post-central, PoCG	L	–60	–18	33	38	3.9647
Inferior parietal gyrus, IPL	L	–36	–42	42	38	5.0662
Superior temporal gyrus, STG	L	–54	–9	0	40	4.9904
Temporal pole gyrus, TPO	L	–51	9	–3	14	4.3405
Middle temporal gyrus, MTG	L	–51	–66	21	45	4.0296
Middle temporal gyrus, MTG	R	63	–18	–12	56	4.0296
**HC < MDD**						
Anterior cingulate cortex, ACC	L	14	30	21	125	–4.3745
Cerebellum, CE	L	–3	–39	–12	28	–4.178
Median cingulate cortex, MCC	R	12	–33	39	66	–4.0981
Middle frontal gyrus, MFG	R	39	9	60	43	4.2547
Orbital inferior frontal gyrus, ORBinf	R	33	–21	9	75	–4.4684
Precentral gyrus, PreCG	R	18	–51	15	40	–6.4008

*MNI, Montreal Neurological Institute; ReHo, regional homogeneity; L, left hemisphere; R, right hemisphere; MDD, major depressive disorder; FDR, false discovery rate. FDR p < 0.05.*

### Gray Matter Volume Results

Gray matter volume was found abnormal in regions of the left ACC (ACC.L), the left superior frontal gyrus (SFG.L), the left middle temporal gyrus (MTG.L), and the ORBinf.R in the MDD and HC groups by ANCOVA (Table 3).

**TABLE 3:** Analysis of covariance of gray matter volume among MDD and HC groups.

ANCOVA results	Hemisphere		*F*	*p*	
Anterior cingulate cortex, ACC	L		7.07	0.008	
Superior frontal gyrus, SFG	L		6.01	0.01	
Middle temporal gyrus, MTG	L		6.77	0.01	
Orbital inferior frontal gyrus, ORBinf	R		8.27	0.005	

**NSI (*n* = 32) < HC (*n* = 69)**	**Hemisphere**	**NSI**	**HC**	**T**	***p***

Anterior cingulate cortex, ACC	L	4,138 ± 396	4,351 ± 467	8.27	0.001
Superior frontal gyrus, SFG	L	1,570 ± 165	1,826 ± 190	6.38	0.003

**MSI (*n* = 64) < HC (*n* = 69)**	**Hemisphere**	**MSI**	**HC**	**T**	***p***

Anterior cingulate cortex, ACC	L	3,858 ± 380	4,351 ± 467	8.70	0.0003
Superior frontal gyrus, SFG	L	1,599 ± 195	1,826 ± 190	5.82	0.004
Orbital inferior frontal gyrus, ORBinf	R	869 ± 107	1,002 ± 178	7.00	0.002

**SSI (*n* = 33) < HC (*n* = 69)**	**Hemisphere**	**SSI**	**HC**	**T**	***p***

Anterior cingulate cortex, ACC	L	3,590 ± 508	4,351 ± 467	10.01	<0.0001
Middle temporal gyrus, MTG	L	6,764 ± 1,360	7,292 ± 1,232	5.81	0.004
Superior frontal gyrus, SFG	L	1,269 ± 187	1,826 ± 190	6.52	0.002
Post-central gyrus, PoCG	L	3,011 ± 613	3,790 ± 558	5.12	0.003
Orbital inferior frontal gyrus, ORBinf	R	821 ± 118	1,002 ± 178	8.43	0.001
Ventral posterior cingulate cortex, vPCC	R	697 ± 148	861 ± 131	4.43	0.012

*The F values were obtained using ANCOVA for age, gender, and education level as covariates.*

*The post hoc t-tests were used to compare among MDD and HC groups.*

*FDR, p < 0.05, healthy group (HC), no suicidal ideation (NSI), mild suicidal ideation (MSI), and severe suicidal ideation (SSI).*

*L, left hemisphere; R, right hemisphere; MDD, major depressive disorder; ANCOVA, analysis of covariance; FDR, false discovery rate.*

Compared with the HC group, the three MDD groups have decreased GMV of the ACC.L and SFG.L. *Post hoc* analysis revealed that GMV of the ACC.L and SFG.L in the NSI group; the ACC.L, SFG.L, and ORBinf.R. in the MSI group; and the ACC.L, MTG.L, SFG.L, PoCG.L, PoCG.R, ORBinf.R, and right ventral posterior cingulate gyrus (vPCC.R) in the SSI group decreased (FDR correction) (Table 3). The GMV of the ACC.L was smaller in the SSI group than in the NSI group (FDR correction) (Figure 2B).

Compared with NSI, GMV of ORBinf.R decreased in SSI and MSI. Compared with MSI, the GMV of ORBinf.R in the SSI group was remarkably reduced (FDR correction) (Figure 2B).

### Structural Covariance Network Results

**FIGURE 3:**
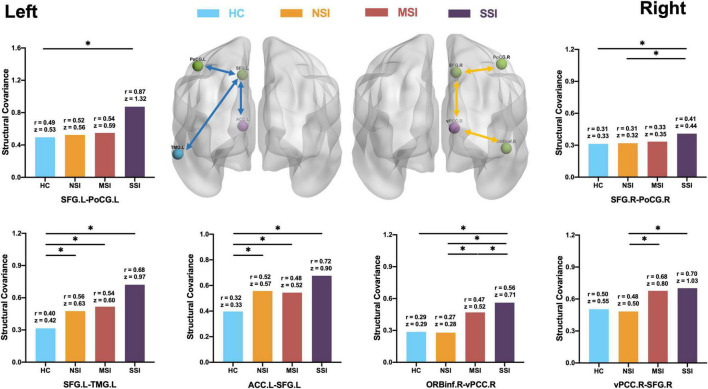
Comparison of CON structural covariance network. **(Left side)** Compared with HC, there is an abnormal increase in the structural covariance of the three MDD groups. The network is concentrated on the left side of the brain. The three pairs include the anterior cingulate gyrus (ACC)–superior frontal gyrus (SFG), SFG–post-central gyrus (PoCG), and SFG–middle temporal gyrus (MTG). **(Right side)** The comparison of the three MDD groups is found to be concentrated on the right side of the brain; three pairs include ORBinf–ventral posterior cingulate gyrus (vPCC), vPCC-SFG, and SFG–PoCG; structural covariance increases with increasing suicidal ideation. There is no difference between HC and NSI. Healthy group (HC), no suicidal ideation (NSI), mild suicidal ideation (MSI), and severe suicidal ideation (SSI). Blue line, comparison between MDD group and HC; yellow line, comparison between MDD three groups. *p < 0.05, false discovery rate (FDR).

Compared with the HC group, SCNs of the ACC.L–SFG.L (HC *r* = 0.32, *z* = 0.33; NSI *r* = 0.52, *z* = 0.57, MSI *r* = 0.48, *z* = 0.52; and SSI *r* = 0.72, *z* = 0.90) and SFG.L–TMG.L (HC *r* = 0.40; *z* = 0.42, NSI *r* = 0.56, *z* = 0.63; MSI *r* = 0.54 *z* = 0.60, and SSI *r* = 0.68, *z* = 0.97) increased in the NSI, MSI, and SSI groups. The SCN of SFG.L–PoCG.L (HC *r* = 0.49, *z* = 0.53; NSI *r* = 0.52, *z* = 0.56; MSI *r* = 0.54, *z* = 0.59 and SSI *r* = 0.87, *z* = 1.32) increased in SSI (FDR *p* < 0.05) (Figure 3, left).

The structural connectivity related to SI is mainly the following three pairs: OrBInf.R–vPCC.R (HC *r* = 0.29, *z* = 0.29; NSI *r* = 0.27, *z* = 0.28, MSI *r* = 0.47, *z* = 0.52 and SSI *r* = 0.56, *z* = 0.71; SSI > MSI > NSI, HC); vPCC.R–SFG.R (HC *r* = 0.50, *z* = 0.55; NSI *r* = 0.48, *z* = 0.50; MSI *r* = 0.68, *z* = 0.80 and SSI *r* = 0.70, *z* = 1.03; SSI, MSI > NSI), and SFG.R–PoCG.R (HC *r* = 0.31, *z* = 0.33; NSI *r* = 0.31, *z* = 0.32; MSI *r* = 0.33, *z* = 0.35 and SSI *r* = 0.41, *z* = 0.44 SSI > MSI, NSI, HC). SCN increased with the increase of Si severity (FDR *p* < 0.05) (Figure 3, right).

## Discussion

Using multimodal imaging analyses, we explored the underlying neuropathological mechanisms associated with SI severity in MDD patients. From analyses of whole-brain ReHo values, we found ReHo changes within the CON. Specifically, ReHo changes of the right middle frontal gyrus and orbital inferior frontal gyrus are negatively correlated with SI severity. Compared to HC, MDD patients were found to have abnormal ReHo and GMV in the ACC.L. GMV of the ORBinf.R was negatively correlated with SI severity. This suggests that there is heterogeneity in the function and structure of CON in MDD patients with SI of different severity. Previous studies have demonstrated intercorrelation between defects in brain structural connection and brain dysfunctions (Honey et al., 2009). Changes in functional dynamics are usually caused by changes in structures; at the same time, long-term functional changes can lead to structural changes through synaptic plasticity (Hagmann et al., 2010). The present study builds upon this structural network analysis and is the first to explore alterations in the structural connectivity of the NSI, MSI, and SSI.

Compared with the HC group, with brain regions with abnormal ReHo located in CON (Dosenbach et al., 2007; Jollant et al., 2011), the MDD patients showed lower ReHo of the ACC.L (Boes et al., 2018). The ACC.L is a key region associated with MDD (Boes et al., 2018; Crowell et al., 2019; Cole et al., 2020; Rappaport et al., 2020). We investigated that the ORBinf.R (*r* = −0.41, *p* < 0.01) was significantly correlated with SI severity (MADRS 10th item, 0–6 scores) at the rest of the MDD group. The ORBinf is a part of the orbitofrontal cortex (OFC) in CON that participates in decision-making, reward learning (Izquierdo, 2017), emotional processes, and cognitive control (Kuusinen et al., 2018). Interestingly, poor decision-making about risk and safety is associated with lateral activation changes in both individuals with SI and their first-degree relatives, indicating that lesions in the OFC (BA47) may be a biomarker of increased risk of suicide (Ding et al., 2016; Johnston et al., 2017). Furthermore, our study also showed that the ReHo values of ORBinf.R in CON are negatively correlated with SI severity.

Compared with the HC group, the MDD patients showed atrophy of the ACC.L, and atrophy of the ACC.L may be related to MDD (Boes et al., 2018). In CON, we found the reduction GMV of the ORBinf.R and decreased GMV with increased SI severity. The magnitude of ORBinf.R volume atrophy and lower ReHo has also been found to be positively correlated with SI score in CON (Yang et al., 2020). A prior analysis showed that reduction in OFC volume (Arnone et al., 2011) and cortical thickness (Suh et al., 2019) in MDD patients was associated with SI (Wagner et al., 2011). The results of the GMV analysis were consistent with the previous ReHo analysis, which also used multimodal imaging analysis to verify the reliability of results. As far as we know, there have been few studies on the structure of the CON focusing on SI at different severity, and most related research has focused on functional abnormalities, which are often due to structural abnormalities (Fried and Nesse, 2015).

Unlike the structural networks of the HC group, network connectivity in the MDD groups was abnormal, and as the severity of illness abnormality spread from the ACC.L to the SFG.L, this then affected the left central posterior gyrus and left middle temporal gyrus; this finding is consistent with a series of studies (Repple et al., 2020; Yang et al., 2020). The cingulate gyrus forms a C-shape adjacent to the prefrontal lobe (Williams(ed.), 2021). As MDD progresses, abnormal information transmission by SFG.L neurons or reduction of regional nutrition results in increased structural connectivity between the two brain regions, and SFG.L is also affected (Raj et al., 2012; Weickenmeier et al., 2018) by shrinkage and abnormal structural connectivity. A functional brain network study of repetitive transcranial magnetic stimulation (TMS) treatment of MDD (Philip et al., 2018) found that the ACC.L of the pathological neural network was connected to the left prefrontal lobe; after receiving TMS treatment, abnormalities of the frontal and parietal network are improved in clinically cured patients (Belleau et al., 2019). Our results on the MDD and HC groups are basically consistent with previous structural and functional network studies, indicating the feasibility of SCN analysis.

In the present study, MDD patients with SI at different severity showed not only specific differences in ReHo and GMV but also characteristic structural connectivity between brain regions in the SCN. Abnormal structural connectivity related to SI was concentrated in the right hemisphere of the CON in three circuits: ORBinf.R–vPCC.R, vPCC.R–SFG.R, and SFG.R–PoCG.R. The concentration in the right hemisphere may be because the right OFC is closely involved in cognitive control and decision-making (Ding et al., 2016; Johnston et al., 2017). Efferent nerve fibers traveling from the OFC to the cingulate gyrus enable it to influence behavior and physiological responses (Burks et al., 2018). As a result, ORBinf.R atrophy (Yang et al., 2020) affects the communication of information between the right-sided CON brain regions, resulting in the spread of vPCC.R abnormalities to the frontal and parietal regions. Eventually, the CON is progressively damaged with SI increases. GMV atrophy of the ORBinf.R and ORBinf.R–vPCC.R circuit abnormality may be associated with SI at different severity and used as a marker for suicide prediction and assessment in the future.

Our findings are consistent with previous studies that demonstrated that MDD is a degenerative mental illness dominated by disrupted ReHo (Späti et al., 2015) and GMV atrophy (Schmaal et al., 2020; Zhang et al., 2020; Stein et al., 2021). By using ReHo and SCN analysis (Lerch et al., 2006; Alexander-Bloch et al., 2013), we found that compared with healthy people, MDD patients had functional and structural defects in the CON and showed disrupted ReHo and GMV atrophy in the ACC.L and abnormal structural connectivity of the ACC.L–SFG.L (Boes et al., 2018; Crowell et al., 2019; Cole et al., 2020; Rappaport et al., 2020). MDD with SI at different severity has specific functional defects and structural network changes located in the ORBinf.R of CON (Yang et al., 2020) and showed disrupted ReHo and GMV atrophy in the ORBinf.R and abnormal structural connectivity of the ORBinf.R–vPCC.R (Kappelmann et al., 2021). Our finding suggested that MDD patients with different severity of SI have different neuroimaging changes.

## Limitations and Future Directions

A significant limitation of this study is its cross-sectional design. A longitudinal study is required to confirm the results. The number of MDD patients with severe SI is small in the present study. Participants in this study were not professionally assessed by SI and were simply grouped according to the MADRS for SI, which should be further analyzed in detail using the professional suicide assessment scale in the future.

## Data Availability Statement

The datasets presented in this article are not readily available because the data are currently part of a longitudinal study that is ongoing. However, they can be obtained by contacting the corresponding author. Requests to access the datasets should be directed to corresponding author XX, xfxu2004@sina.com.

## Ethics Statement

The studies involving human participants were reviewed and approved by the Ethics Review Board of Kunming Medical College First Affiliated Hospital [Ethics Review L No. 50(2016)]. The patients/participants provided their written informed consent to participate in this study.

## Author Contributions

XX and ZS participated in the conception and design, data analysis, and interpretation of the research. MH contributed to the conception and design of the project, data collection and analysis, and the writing and revision of the manuscript. LP, ZC, and CZ contributed to data analysis. All authors critically revised the draft version of the manuscript and approved the final manuscript.

## Conflict of Interest

The authors declare that the research was conducted in the absence of any commercial or financial relationships that could be construed as a potential conflict of interest.

## Publisher’s Note

All claims expressed in this article are solely those of the authors and do not necessarily represent those of their affiliated organizations, or those of the publisher, the editors and the reviewers. Any product that may be evaluated in this article, or claim that may be made by its manufacturer, is not guaranteed or endorsed by the publisher.

## Footnotes

1
http://rfmri.org/DPABI

2
https://surfer.nmr.mgh.harvard.edu

3
http://restfmri.net/forum/REST_V1.8
