# Supplementary material for: Identifying Changes of Brain Regional Homogeneity and Cingulo-Opercular Network Connectivity in First-Episode, Drug-Naïve Depressive Patients With Suicidal Ideation
Source: Front Neurosci. 2022 Mar 2;16:856366. doi: 10.3389/fnins.2022.856366 (PMC8924659; doi:10.3389/fnins.2022.856366)
Supplement: Supplementary file 1 [file Data_Sheet_1.docx]

**Supplementary Table1. Compared with HC, NSI group gray matter volume (GMV) in CON（58 brain regions）**

|  |  | **HC** | | **NSI** | |  |
| --- | --- | --- | --- | --- | --- | --- |
| **Region** | **Hemisphere** | **Mean** | **SD** | **Mean** | **SD** | **P** |
| **Paracentral lobules, PCL** | **L** | 2715.9 | 301.2 | 2415.9 | 361.1 | 0.399 |
|  | **R** | 3019.0 | 412.8 | 2918.2 | 412.2 | 0.933 |
| **Subcentral, Subc** | **L** | 3386.0 | 260.1 | 3041.7 | 274.4 | 0.059 |
|  | **R** | 3381.2 | 256.3 | 3479.9 | 251.0 | 0.018^b^ |
| **Anterior cingulate cortex, ACC** | **L** | 4351.2 | 467.3 | 4137.7 | 395.9 | **0.001^a^** |
|  | **R** | 3186.2 | 491.3 | 2902.9 | 401.4 | 0.142 |
| **Dorsal posterior cingulate cortex, dPCC** | **L** | 848.5 | 127.5 | 816.3 | 100.6 | 0.034 |
|  | **R** | 898.0 | 220.4 | 713.0 | 226.3 | 0.045 |
| **Ventral posterior cingulate cortex, vPCC** | **L** | 881.7 | 118.8 | 847.8 | 153.7 | 0.610 |
|  | **R** | 861.1 | 131.5 | 880.1 | 115.7 | 0.659 |
| **Cuneus, CUN** | **L** | 14344.9 | 278.9 | 15404.6 | 295.0 | 0.838 |
|  | **R** | 1416.0 | 266.5 | 1469.4 | 183.5 | 0.551 |
| **opercular inferior frontal gyrus, IFGoperc** | **L** | 2060.7 | 322.4 | 2153.6 | 248.1 | 0.134 |
|  | **R** | 3416.5 | 1084.8 | 5699.0 | 788.1 | 0.326 |
| **Orbital inferior frontal gyrus, ORBinf** | **L** | 1090.7 | 201.0 | 1090.4 | 195.3 | 0.049 ^b^ |
|  | **R** | 1001.3 | 177.5 | 969.0 | 166.0 | 0.063 |
| **triangular inferior frontal gyrus, IFGtriang** | **L** | 3302.5 | 456.9 | 3379.3 | 650.4 | 0.330 |
|  | **R** | 3071.8 | 704.7 | 5639.7 | 690.8 | 0.134 |
| **Middle frontal gyrus, IFGtriang** | **L** | 4926.0 | 847.6 | 5513.2 | 771.1 | 0.127 |
|  | **R** | 4771.9 | 630.2 | 4333.1 | 890.2 | 0.008 ^b^ |
| **Superior frontal gyrus, SFG** | **L** | 1826.1 | 190.2 | 1570.0 | 165.0 | **0.003 ^a^** |
|  | **R** | 1708.8 | 313.8 | 1552.3 | 341.5 | 0.017 ^b^ |
| **Long insular gyrus, cINS** | **L** | 6225.4 | 921.3 | 6722.0 | 1173.9 | 0.083 |
|  | **R** | 7388.8 | 1031.7 | 6814.3 | 1026.3 | 0.026 ^b^ |
| **Short insular gyrus, sINS** | **L** | 2515.9 | 301.2 | 2415.9 | 361.1 | 0.008 ^b^ |
|  | **R** | 2819.0 | 412.8 | 3018.2 | 412.2 | 0.079 |
| **Angular gyrus, ANG** | **L** | 6386.0 | 360.1 | 4041.7 | 274.4 | 0.688 |
|  | **R** | 6381.2 | 256.3 | 5479.9 | 251.0 | 0.271 |
| **Supramarginal gyrus, SMG** | **L** | 3811.2 | 148.3 | 2669.7 | 110.9 | 0.390 |
|  | **R** | 3186.2 | 491.3 | 2902.9 | 401.4 | 0.368 |
| **superior parietal lobule, SPG** | **L** | 3898.0 | 620.4 | 3016.3 | 500.6 | 0.324 |
|  | **R** | 3790.2 | 557.8 | 3013.0 | 226.3 | 0.189 |
| **Postcentral gyrus, PoCG** | **L** | 3111.1 | 303.5 | 3047.8 | 553.7 | 0.049 ^b^ |
|  | **R** | 4344.9 | 478.9 | 3880.1 | 415.7 | 0.162 |
| **Precentral gyrus, PreCG** | **L** | 4160.0 | 566.5 | 15404.6 | 595.0 | 0.017 ^b^ |
|  | **R** | 3898.0 | 620.4 | 4692.4 | 583.5 | 0.029 ^b^ |
| **Precuneus, PCUN** | **L** | 5060.7 | 722.4 | 2153.6 | 248.1 | 0.244 |
|  | **R** | 6416.5 | 884.8 | 5699.0 | 788.1 | 0.158 |
| **Superior temporal gyrus (lateral aspect), TSL** | **L** | 5078.7 | 826.0 | 5944.4 | 995.3 | 0.128 |
|  | **R** | 4652.3 | 605.2 | 5684.0 | 714.4 | 0.124 |
| **Superior temporal gyrus (planum polare), TPOsup** | **L** | 2302.5 | 256.9 | 3679.3 | 650.4 | 0.613 |
|  | **R** | 2671.8 | 304.7 | 5639.7 | 690.8 | 0.830 |
| **Superior temporal gyrus (planum temporale),TSPt** | **L** | 5926.0 | 847.6 | 5513.2 | 771.1 | 0.693 |
|  | **R** | 4771.9 | 630.2 | 5333.1 | 890.2 | 0.140 |
| **Inferior temporal gyrus, ITG** | **L** | 7116.7 | 1237.2 | 1957.0 | 391.0 | 0.440 |
|  | **R** | 6508.8 | 1124.8 | 1752.3 | 341.5 | 0.158 |
| **Middle temporal gyrus, MTG** | **L** | 7292.4 | 1232.3 | 6722.0 | 1173.9 | 0.007 ^b^ |
|  | **R** | 7388.8 | 1031.7 | 6814.3 | 1026.3 | 0.018 ^b^ |
| **Thalamus, Tha** | **L** | 7248.3 | 443.4 | 7195.5 | 713.4 | 0.144 |
|  | **R** | 6821.8 | 347.5 | 6691.0 | 699.9 | 0.037 ^b^ |
| **Caudate nucleus, Ca** | **L** | 3523.1 | 355.0 | 3371.0 | 450.3 | 0.705 |
|  | **R** | 3580.1 | 385.9 | 3686.7 | 491.3 | 0.307 |
| **Putamen, Pu** | **L** | 2400.1 | 151.0 | 5205.9 | 513.6 | 0.343 |
|  | **R** | 2310.5 | 239.9 | 5310.5 | 576.5 | 0.664 |
| **globus pallidum, GP** | **L** | 2985.0 | 185.3 | 1940.5 | 272.2 | 0.034 ^b^ |
|  | **R** | 2037.8 | 204.7 | 2017.9 | 244.4 | 0.161 |
| **nucleus accumbent, NAcc** | **L** | 647.2 | 88.8 | 609.5 | 104.0 | 0.059 |
|  | **R** | 617.1 | 108.4 | 616.1 | 104.3 | 0.962 |

^a^ : FDR, p <0.05;

^b^: NOT correction p <0.05;

L, left hemisphere; R, right hemisphere.

The p values were obtained using two-way analysis of covariance adjusted for age, gender, and education level as covariates.

**Supplementary Table2. Compared with HC, MSI group gray matter volume (GMV) in CON（58 brain regions）**

|  |  | **HC** | | **MSI** | |  |
| --- | --- | --- | --- | --- | --- | --- |
| **Region** | **Hemisphere** | **Mean** | **SD** | **Mean** | **SD** | **P** |
| **Paracentral lobules, PCL** | **L** | 2715.9 | 301.2 | 2775.5 | 369.6 | 0.960 |
|  | **R** | 3019.0 | 412.8 | 2381.8 | 325.4 | 0.470 |
| **Subcentral, Subc** | **L** | 3386.0 | 560.1 | 3177.3 | 618.3 | 0.818 |
|  | **R** | 3381.2 | 456.3 | 3031.1 | 568.6 | 0.983 |
| **Anterior cingulate cortex, ACC** | **L** | 4351.2 | 467.3 | 3858.1 | 380.3 | **0.0003^a^** |
|  | **R** | 3186.2 | 491.3 | 3041.9 | 362.6 | 0.764 |
| **Dorsal posterior cingulate cortex, dPCC** | **L** | 848.5 | 127.5 | 804.4 | 210.4 | 0.975 |
|  | **R** | 898.0 | 220.4 | 872.1 | 263.5 | 0.621 |
| **Ventral posterior cingulate cortex, vPCC** | **L** | 881.7 | 118.8 | 683.4 | 141.1 | 0.960 |
|  | **R** | 861.1 | 131.5 | 811.2 | 175.3 | 0.636 |
| **Cuneus, CUN** | **L** | 14344.9 | 278.9 | 1940.2 | 232.9 | 0.788 |
|  | **R** | 1416.0 | 266.5 | 1179.1 | 318.9 | 0.443 |
| **opercular inferior frontal gyrus, IFGoperc** | **L** | 2060.7 | 322.4 | 3225.0 | 630.7 | 0.706 |
|  | **R** | 3416.5 | 1084.8 | 3109.6 | 511.8 | 0.648 |
| **Orbital inferior frontal gyrus, ORBinf** | **L** | 1090.7 | 201.0 | 1078.2 | 197.3 | 0.596 |
|  | **R** | 1001.3 | 177.5 | 868.9 | 106.5 | **0.002 ^a^** |
| **triangular inferior frontal gyrus, IFGtriang** | **L** | 3302.5 | 456.9 | 2527.6 | 591.5 | 0.803 |
|  | **R** | 3071.8 | 704.7 | 2034.1 | 467.7 | 0.003^b^ |
| **Middle frontal gyrus, IFGtriang** | **L** | 4926.0 | 847.6 | 4084.3 | 419.4 | 0.363 |
|  | **R** | 4771.9 | 630.2 | 4586.2 | 505.2 | 0.987 |
| **Superior frontal gyrus, SFG** | **L** | 1826.1 | 190.2 | 1598.8 | 194.7 | **0.004 ^a^** |
|  | **R** | 1708.8 | 313.8 | 1578.1 | 173.0 | 0.161 |
| **Long insular gyrus, cINS** | **L** | 6225.4 | 921.3 | 6517.9 | 822.3 | 0.487 |
|  | **R** | 7388.8 | 1031.7 | 6477.5 | 849.7 | 0.143 |
| **Short insular gyrus, sINS** | **L** | 2515.9 | 301.2 | 2285.2 | 266.8 | 0.951 |
|  | **R** | 2819.0 | 412.8 | 2150.7 | 379.2 | 0.602 |
| **Angular gyrus, ANG** | **L** | 6386.0 | 360.1 | 5867.8 | 347.2 | 0.591 |
|  | **R** | 6381.2 | 256.3 | 5564.2 | 299.3 | 0.025 ^b^ |
| **Supramarginal gyrus, SMG** | **L** | 3811.2 | 148.3 | 3307.9 | 204.7 | 0.359 |
|  | **R** | 3186.2 | 191.3 | 3421.6 | 253.7 | 0.238 |
| **superior parietal lobule, SPG** | **L** | 3048.5 | 227.5 | 2730.0 | 212.9 | 0.414 |
|  | **R** | 3898.0 | 620.4 | 3728.3 | 713.6 | 0.461 |
| **Postcentral gyrus, PoCG** | **L** | 3790.2 | 557.8 | 3344.5 | 623.3 | 0.655 |
|  | **R** | 3111.1 | 303.5 | 3051.6 | 457.1 | 0.999 |
| **Precentral gyrus, PreCG** | **L** | 4344.9 | 478.9 | 4058.2 | 515.5 | 0.015 ^b^ |
|  | **R** | 4160.0 | 266.5 | 5916.8 | 765.4 | 0.534 |
| **Precuneus, PCUN** | **L** | 5060.7 | 722.4 | 5075.5 | 777.5 | 0.831 |
|  | **R** | 6416.5 | 884.8 | 5136.4 | 783.5 | 0.829 |
| **Superior temporal gyrus (lateral aspect), TSL** | **L** | 5078.7 | 826.0 | 5242.0 | 821.6 | 0.287 |
|  | **R** | 4652.3 | 605.2 | 4360.1 | 660.1 | 0.328 |
| **Superior temporal gyrus (planum polare), TPOsup** | **L** | 2302.5 | 256.9 | 2031.0 | 350.7 | 0.594 |
|  | **R** | 2671.8 | 304.7 | 2090.1 | 353.6 | 0.845 |
| **Superior temporal gyrus (planum temporale),TSPt** | **L** | 5926.0 | 847.6 | 4792.9 | 514.9 | 0.896 |
|  | **R** | 4771.9 | 630.2 | 4581.2 | 795.6 | 0.564 |
| **Inferior temporal gyrus, ITG** | **L** | 7116.7 | 1237.2 | 7098.9 | 1331.3 | 0.460 |
|  | **R** | 6508.8 | 1124.8 | 6503.9 | 1160.1 | 0.900 |
| **Middle temporal gyrus, MTG** | **L** | 7292.4 | 1232.3 | 6293.2 | 1283.1 | 0.998 |
|  | **R** | 7388.8 | 1031.7 | 6836.6 | 1210.6 | 0.099 ^b^ |
| **Thalamus, Tha** | **L** | 7248.3 | 443.4 | 7731.8 | 380.5 | 0.527 |
|  | **R** | 6821.8 | 347.5 | 6731.6 | 343.9 | 0.914 |
| **Caudate nucleus, Ca** | **L** | 3523.1 | 355.0 | 3222.8 | 218.1 | 0.846 |
|  | **R** | 3580.1 | 385.9 | 3741.8 | 311.4 | 0.481 |
| **Putamen, Pu** | **L** | 2400.1 | 151.0 | 1934.5 | 205.4 | 0.142 |
|  | **R** | 2310.5 | 239.9 | 2023.9 | 249.4 | 0.730 |
| **globus pallidum, GP** | **L** | 2985.0 | 185.3 | 2915.0 | 173.3 | 0.029 ^b^ |
|  | **R** | 2037.8 | 204.7 | 2041.0 | 122.9 | 0.081 |
| **nucleus accumbent, NAcc** | **L** | 647.2 | 88.8 | 621.3 | 104.4 | 0.132 |
|  | **R** | 617.1 | 108.4 | 569.0 | 89.5 | 0.005 ^b^ |

^a^ : FDR, p <0.05;

^b^: NOT correction p <0.05;

L, left hemisphere; R, right hemisphere.

The p values were obtained using two-way analysis of covariance adjusted for age, gender, and education level as covariates.

**Supplementary Table3. Compared with HC, SSI group gray matter volume (GMV) in CON（58 brain regions）**

|  |  | **HC** | | **SSI** | |  |
| --- | --- | --- | --- | --- | --- | --- |
| **Region** | **Hemisphere** | **Mean** | **SD** | **Mean** | **SD** | **P** |
| **Paracentral lobules, PCL** | **L** | 2715.9 | 301.2 | 2767.5 | 333.6 | 0.563 |
|  | **R** | 3019.0 | 412.8 | 2333.8 | 313.4 | 0.350 |
| **Subcentral, Subc** | **L** | 3386.0 | 560.1 | 3177.3 | 618.3 | 0.413 |
|  | **R** | 3381.2 | 456.3 | 3001.9 | 459.9 | 0.482 |
| **Anterior cingulate cortex, ACC** | **L** | 4351.2 | 467.3 | 3590.1 | 444.1 | **<0.0001^a^** |
|  | **R** | 3186.2 | 491.3 | 3011.2 | 461.6 | 0.038 ^b^ |
| **Dorsal posterior cingulate cortex, dPCC** | **L** | 848.5 | 127.5 | 724.8 | 240.1 | 0.770 |
|  | **R** | 898.0 | 220.4 | 767.1 | 233.2 | 0.340 |
| **Ventral posterior cingulate cortex, vPCC** | **L** | 881.7 | 118.8 | 773.4 | 151.6 | 0.040 ^b^ |
|  | **R** | 861.1 | 131.5 | 697.2 | 131.2 | 0.012 ^b^ |
| **Cuneus, CUN** | **L** | 14344.9 | 278.9 | 1712.8 | 302.1 | 0.937 |
|  | **R** | 1416.0 | 266.5 | 1329.1 | 278.7 | 0.671 |
| **opercular inferior frontal gyrus, IFGoperc** | **L** | 2060.7 | 322.4 | 2234.3 | 523.8 | 0.906 |
|  | **R** | 3416.5 | 1084.8 | 3451.1 | 531.1 | 0.564 |
| **Orbital inferior frontal gyrus, ORBinf** | **L** | 1090.7 | 201.0 | 1039.9 | 204.7 | 0.478 |
|  | **R** | 1001.3 | 177.5 | 868.9 | 106.5 | **0.002 ^a^** |
| **triangular inferior frontal gyrus, IFGtriang** | **L** | 3302.5 | 456.9 | 2498.1 | 394.6 | 0.611 |
|  | **R** | 3071.8 | 704.7 | 2134.1 | 607.3 | 0.002^b^ |
| **Middle frontal gyrus, IFGtriang** | **L** | 4926.0 | 847.6 | 4345.7 | 422.4 | 0.263 |
|  | **R** | 4771.9 | 630.2 | 4456.8 | 412.7 | 0.423 |
| **Superior frontal gyrus, SFG** | **L** | 1826.1 | 190.2 | 1598.8 | 194.7 | **0.004 ^a^** |
|  | **R** | 1708.8 | 313.8 | 1588.0 | 201.7 | 0.060 |
| **Long insular gyrus, cINS** | **L** | 6225.4 | 921.3 | 6245.9 | 801.3 | 0.872 |
|  | **R** | 7388.8 | 1031.7 | 6473.1 | 817.1 | 0.056 |
| **Short insular gyrus, sINS** | **L** | 2515.9 | 301.2 | 2341.1 | 245.6 | 0.341 |
|  | **R** | 2819.0 | 412.8 | 2819.0 | 288.0 | 0.856 |
| **Angular gyrus, ANG** | **L** | 6386.0 | 360.1 | 5989.1 | 107.3 | 0.521 |
|  | **R** | 6381.2 | 256.3 | 5430.4 | 180.0 | 0.021 ^b^ |
| **Supramarginal gyrus, SMG** | **L** | 3811.2 | 148.3 | 3213.9 | 213.1 | 0.341 |
|  | **R** | 3186.2 | 191.3 | 3031.1 | 167.9 | 0.100 |
| **superior parietal lobule, SPG** | **L** | 3048.5 | 227.5 | 3030.0 | 312.4 | 0.370 |
|  | **R** | 3898.0 | 620.4 | 3890.3 | 624.8 | 0.458 |
| **Postcentral gyrus, PoCG** | **L** | 3790.2 | 557.8 | 3011.1 | 612.7 | **0.003^a^** |
|  | **R** | 3111.1 | 303.5 | 3100.6 | 206.8 | 0.100 |
| **Precentral gyrus, PreCG** | **L** | 4344.9 | 478.9 | 4001.9 | 412.7 | 0.005^b^ |
|  | **R** | 4160.0 | 266.5 | 4302.8 | 765.4 | 0.134 |
| **Precuneus, PCUN** | **L** | 5060.7 | 722.4 | 5002.5 | 678.1 | 0.031 ^b^ |
|  | **R** | 6416.5 | 884.8 | 6114.5 | 802.1 | 0.012 ^b^ |
| **Superior temporal gyrus (lateral aspect), TSL** | **L** | 5078.7 | 826.0 | 5066.6 | 817.0 | 0.232 |
|  | **R** | 4652.3 | 605.2 | 4123.3 | 630.2 | 0.312 |
| **Superior temporal gyrus (planum polare), TPOsup** | **L** | 2302.5 | 256.9 | 2310.3 | 290.0 | 0.500 |
|  | **R** | 2671.8 | 304.7 | 2001.9 | 297.7 | 0.019 ^b^ |
| **Superior temporal gyrus (planum temporale),TSPt** | **L** | 5926.0 | 847.6 | 4526.1 | 524.1 | 0.451 |
|  | **R** | 4771.9 | 630.2 | 4386.7 | 651.4 | 0.431 |
| **Inferior temporal gyrus, ITG** | **L** | 7116.7 | 1237.2 | 7101.4 | 1131.1 | 0.320 |
|  | **R** | 6508.8 | 1124.8 | 6410.0 | 1140.3 | 0.781 |
| **Middle temporal gyrus, MTG** | **L** | 7292.4 | 1232.3 | 6764.2 | 1360.4 | **0.004^a^** |
|  | **R** | 7388.8 | 1031.7 | 6766.1 | 1110.0 | 0.070 |
| **Thalamus, Tha** | **L** | 7248.3 | 443.4 | 7012.3 | 375.2 | 0.620 |
|  | **R** | 6821.8 | 347.5 | 6349.0 | 323.9 | 0.123 |
| **Caudate nucleus, Ca** | **L** | 3523.1 | 355.0 | 3145.6 | 222.7 | 0.980 |
|  | **R** | 3580.1 | 385.9 | 3330.9 | 301.2 | 0.371 |
| **Putamen, Pu** | **L** | 2400.1 | 151.0 | 2001,7 | 212.4 | 0.106 |
|  | **R** | 2310.5 | 239.9 | 2013.4 | 225.2 | 0.452 |
| **globus pallidum, GP** | **L** | 2985.0 | 185.3 | 2789.1 | 170.5 | 0.008 ^b^ |
|  | **R** | 2037.8 | 204.7 | 2041.3 | 187.9 | 0.081 |
| **nucleus accumbent, NAcc** | **L** | 647.2 | 88.8 | 601.9 | 87.9 | 0.310 |
|  | **R** | 617.1 | 108.4 | 576.1 | 90.7 | 0.004 ^b^ |

^a^ : FDR, p <0.05;

^b^: NOT correction p <0.05;

L, left hemisphere; R, right hemisphere.

The p values were obtained using two-way analysis of covariance adjusted for age, gender, and education level as covariates.
